# Complex Interaction Between Low-Frequency APD Oscillations and Beat-to-Beat APD Variability in Humans Is Governed by the Sympathetic Nervous System

**DOI:** 10.3389/fphys.2019.01582

**Published:** 2020-01-22

**Authors:** Stefan Van Duijvenboden, Bradley Porter, Esther Pueyo, David Adolfo Sampedro-Puente, Jesus Fernandez-Bes, Baldeep Sidhu, Justin Gould, Michele Orini, Martin J. Bishop, Ben Hanson, Pier Lambiase, Reza Razavi, Christopher A. Rinaldi, Jaswinder S. Gill, Peter Taggart

**Affiliations:** ^1^Institute of Cardiovascular Science, University College London, London, United Kingdom; ^2^School of Imaging Sciences and Biomedical Engineering, King’s College London, London, United Kingdom; ^3^BSICOS Group, I3A, IIS Aragón, University of Zaragoza, Zaragoza, Spain; ^4^CIBER-BBN, Madrid, Spain; ^5^Department of Clinical Pharmacology, Queen Mary University of London, London, United Kingdom; ^6^Department of Mechanical Engineering, University College London, London, United Kingdom; ^7^Guy’s and St Thomas’ NHS Foundation Trust, London, United Kingdom

**Keywords:** action potential duration, beat-to-beat variability, oscillations, human heart, sympathetic, beta-adrenergic blockade

## Abstract

**Background:**

Recent clinical, experimental and modeling studies link oscillations of ventricular repolarization in the low frequency (LF) (approx. 0.1 Hz) to arrhythmogenesis. Sympathetic provocation has been shown to enhance both LF oscillations of action potential duration (APD) and beat-to-beat variability (BVR) in humans. We hypothesized that beta-adrenergic blockade would reduce LF oscillations of APD and BVR of APD in humans and that the two processes might be linked.

**Methods and Results:**

Twelve patients with normal ventricles were studied during routine electrophysiological procedures. Activation-recovery intervals (ARI) as a conventional surrogate for APD were recorded from 10 left and 10 right ventricular endocardial sites before and after acute beta-adrenergic adrenergic blockade. Cycle length was maintained constant with right ventricular pacing. Oscillatory behavior of ARI was quantified by spectral analysis and BVR as the short-term variability. Beta-adrenergic blockade reduced LF ARI oscillations (8.6 ± 4.5 ms^2^ vs. 5.5 ± 3.5 ms^2^, *p* = 0.027). A significant correlation was present between the initial control values and reduction seen following beta-adrenergic blockade in LF ARI (*r*_s_ = 0.62, *p* = 0.037) such that when initial values are high the effect is greater. A similar relationship was also seen in the beat-to beat variability of ARI (*r*_s_ = 0.74, *p* = 0.008). There was a significant correlation between the beta-adrenergic blockade induced reduction in LF power of ARI and the witnessed reduction of beat-to-beat variability of ARI (*r*_s_ = 0.74, *p* = 0.01). These clinical results accord with recent computational modeling studies which provide mechanistic insight into the interactions of LF oscillations and beat-to-beat variability of APD at the cellular level.

**Conclusion:**

Beta-adrenergic blockade reduces LF oscillatory behavior of APD (ARI) in humans *in vivo*. Our results support the importance of LF oscillations in modulating the response of BVR to beta-adrenergic blockers, suggesting that LF oscillations may play role in modulating beta-adrenergic mechanisms underlying BVR.

## Introduction

Factors which influence the stability of ventricular repolarization are important in arrhythmogenesis. Enhanced oscillation of ventricular repolarization in the low frequency range and increased beat-to-beat variability (BVR) of ventricular repolarization are two of the strongest predictors of arrhythmia and sudden cardiac death (Atiga et al., 1998; Haigney et al., 2004; Thomsen et al., 2004; Gallacher et al., 2007; Tereshchenko et al., 2009; Abi-Gerges et al., 2010; Hinterseer et al., 2010; Jacobson et al., 2011; Średniawa et al., 2012; Rizas et al., 2014, 2016, 2017; Baumert et al., 2016; Bauer et al., 2019). Both are enhanced by sympathetic stimulation and recent studies suggest a possible interactive mechanism (Porter et al., 2018). However, the mechanisms underlying the effect of beta-adrenergic stimulation on LF oscillations of repolarization and beat-to-beat variability of repolarization remain unclear.

Oscillations of ventricular repolarization measured from the ECG T-wave vector referred to as periodic repolarisation dynamics (PRD) have been attributed to oscillations in APD at the frequency of the sympathetic nerves (approx. 0.05–0.1 Hz). Ventricular action potential duration (APD) measured as activation-recovery intervals (ARI) has recently been shown to oscillate in this frequency range (Hanson et al., 2014). The LF power of APD has been shown to be increased by sympathetic provocation (Porter et al., 2018). The recent finding of LF oscillations in short term variability of ventricular APD (Porter et al., 2018) raises the possibility of an association between LF oscillations of APD and BVR.

Computational modeling has provided early insight into the mechanisms underlying these oscillations of APD, the effect of beta-adrenergic stimulation and their relationship to the initiation of ventricular arrhythmias (Pueyo et al., 2016a, b). More recent studies on the effect of beta-adrenergic blockade suggest that the cellular mechanisms underlying modulation of LF APD and BVR of APD are strongly influenced by the initial conditions of APD (Sampedro-Puente et al., 2019). One of the objectives of the present study was to examine this hypothesis in humans *in vivo*.

We have studied 12 patients during cardiac catheterization allowing us to measure ARIs as an approximation for APD at 10 right ventricular (RV) and 10 left ventricular (LV) endocardial sites in order to investigate the effect of acute beta-adrenergic blockade on LF oscillations of ventricular APD and on BVR of APD, and the possible interaction between the two. Cycle length was held constant with RV pacing to avoid confounding effects due to the cycle length dependency of APD.

## Materials and Methods

### Ethical Approval

The study was approved by the Ethics Committee of Guy’s and Thomas’ Hospitals and conformed to the standards set by the Declaration of Helsinki (latest revision: 59th World Medical Association General Assembly). All patients gave written, informed consent.

### Subjects

Studies were performed in 12 patients (10 males, 2 females, aged 41–69, median 61) during the course of routine clinical radiofrequency ablation procedures for atrial fibrillation. Four patients had paroxysmal atrial fibrillation, and eight patients had persistent atrial fibrillation. All subjects had normal biventricular systolic function. Table 1 demonstrates further patient characteristics. Studies were performed in the un-sedated state and cardio-active medications (beta-blockers, non-dihydropyridine calcium channel blockers, digoxin, and flecainide) were discontinued for 5 days before the study.

**TABLE 1:** Patient characteristics.

Diabetes	2(17%)
Sleep apnoea	0(0%)
Hypertension	5(42%)
Left atrial diameter	4.2±0.4 cm
Presence of left ventricular hypertrophy	2(17%)
Presence of diastolic dysfunction	2(17%)
Beta-blocker	7(60%)
Non-dihydropyridine calcium channel blocker	1(8%)
Amiodarone	0(0%)
Digoxin	1(8%)
Flecainide	1(8%)

### Protocol

**FIGURE 1:**
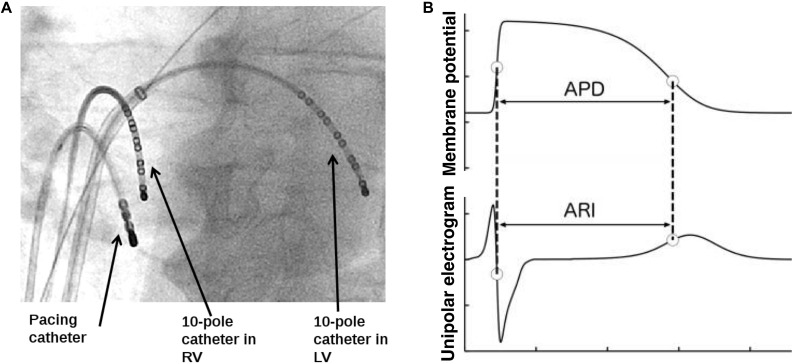
Diagram of **(A)** decapolar catheter electrodes in RV and LV and pacing wire and **(B)** the schematic illustration of relation between the activation recovery interval (ARI) in the unipolar EGM and the ventricular action potential duration (APD).

Utilizsing the routine transseptal puncture of an AF ablation, a decapolar catheter was placed in the left ventricle via the left atrium and mitral valve. The pacing catheter and second decapolar catheter were placed in the right ventricle. Routine AF ablation femoral venous access was utilized for placement of all catheters. The transseptal puncture was conducted under radiographic guidance. Figure 1A shows the set-up of both recording decapolar catheters and the pacing catheter. Subjects were paced from the right ventricular apex using a Biotronik (Berlin, Germany) stimulator (model UHS 3000) at 2x diastolic threshold and 2 ms pulse width, at a cycle length >20 beats/min faster than the intrinsic AF rate (median: 500 ms; range: 360–500 ms) to avoid breakthrough of intrinsic beats. A 2-min period of adaptation to the paced cycle length was applied before starting a controlled breathing protocol. Breathing was controlled throughout the protocol at 0.25 and 0.5 Hz. Recordings took place for 90s during each controlled breathing cycle. First, a control period was established with the breathing protocol performed in absence of any autonomic blocking agents. Pacing was then stopped and the subject received metoprolol at a dose sufficient to reduce the intrinsic heart rate by 10 beats/min (iv; dose range, 2–10 mg), and after a further 10 min for equilibration the pacing (at the same paced cycle length as the control) and breathing protocol was repeated as above. The entire study protocol was completed prior to conducting the AF ablation.

### Measurements

Continuous synchronous recordings of femoral arterial blood pressure and unipolar electrograms (UEGs) at 10 endocardial RV and 10 endocardial LV sites (Figure 1A) were obtained before routine clinical radiofrequency ablation procedures for atrial fibrillation in the cardiac catheterization lab at St Thomas’ Hospital in London, as described previously (Hanson et al., 2012; van Duijvenboden et al., 2015). UEGs and blood pressure recordings were digitized at 1,200 Hz (Ensite 3000; Endocardial Solutions) and analyzed offline.

### Data Analysis

UEGs were analyzed for ventricular APDs at each recording site by measuring activation-recovery intervals (ARIs) using the Wyatt method (Wyatt et al., 1981). This method has been validated in theoretical, computational, and experimental studies (Wyatt et al., 1981; Haws and Lux, 1990; Coronel et al., 2006; Potse et al., 2009). According to this method, activation is measured at the moment of minimum dV/dt of the QRS complex of the UEG and repolarization at the moment of maximum dV/dt of the T-wave (Figure 1B). ARIs were measured automatically using in house developed algorithms. Heuristic-based screening was used to identify and discount any cases where the T-wave was indistinct or corrupt. Blood pressure recordings were analyzed for systolic blood pressure (SBP) and the maximum rate of systolic pressure increase (dP/dt_max_) as a measure of myocardial contractility. Measurement of dP/dt_max_ from the femoral artery has been shown to provide good tracking of left ventricular contractility (Monge Garcia et al., 2018).

To establish evenly sampled series, any beats for which ARI, SBP or dP/dt_max_ measurements could not be determined were replaced by linear interpolation between the surrounding beats. Recordings were rejected from the analysis if these surrogate beats constituted more than 10% of any series.

The low frequency (LF) power in each ARI series was estimated by calculating the bandpower in the low-frequency band (0.04–0.15 Hz) using the Thomson’s multitaper method with three Slepian tapers, which is known to be robust against noise (Thomson, 1982). The same analysis was applied to the calculate the high frequency (HF) power in in a frequency band of the breathing frequency (either 0.25 or 0.5 Hz) ± 10%. The LF and HF powers were then averaged for RV and LV poles.

Beat-to-beat variability of ARI was assessed by computing the short term variability (STV) of ARIs for each endocardial recording site over the entire recording as per established STV measures (Johnson et al., 2013; Baumert et al., 2016). The STV ARI (STV-ARI) was computed using a moving window of 10 consecutive beats:

$$\mathrm{STV}=\frac{\sum \left|{\mathrm{ARI}}_{i-1}-{\mathrm{ARI}}_{i}\right|}{N\,\sqrt{2}}$$

where *ARI_i_* is the ARI at the *i*th beat and N is the number of beats. For each pole, we computed the mean STV in time and then averaged these values across poles. The STV of SBP (STV-SBP) and dP/dt_max_ (STV-dP/dt_max_) were computed using the same formula and number of beats as for ARI.

### Statistical Analysis

Results were averaged across the two separate breathing cycles for both control recordings and following introduction of beta-adrenergic blockade. Results are presented as mean ± standard deviation for normally distributed variables and as median and interquartile range (IQR) for non-normally distributed variables. The effect of beta-adrenergic blockade on LF power for ARI, SBP and dP/dt_max_ was tested for statistical significance using the two-tailed paired Wilcoxon signed–rank test. To evaluate whether there were different responses in ARI STV between individual electrodes (*n* = 20), we used the non-parametric Kruskal–Wallis test. Results were considered significant at *p* < 0.05.

## Results

### Effect of Beta-Adrenergic Blockade on Combined Group Data

**FIGURE 2:**
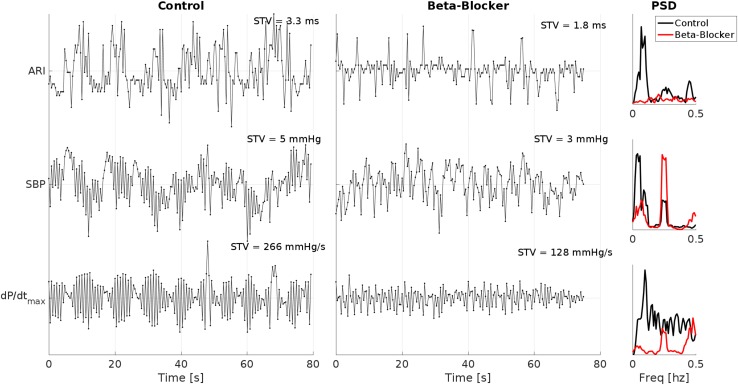
Example ARI, SBP and dP/dt_max_ time series of one patient breathing at 15 breaths/min (0.25 Hz) during control and following beta-adrenergic blockade. Clear low-frequency oscillations are visible in all traces during control, which are attenuated following beta-adrenergic blockade. Also note the reduction in the short time variability (STV) measures. PSD, power spectral density.

Example ARI, SBP and dP/dt_max_ time series of one patient breathing at 15 breaths/min (0.25 Hz) during control and following beta-adrenergic blockade are shown in Figure 2. In this example, clear LF oscillations are visible in all traces during control. which are attenuated following beta-adrenergic blockade. At the same time, there is a clear reduction in the STV.

**FIGURE 3:**
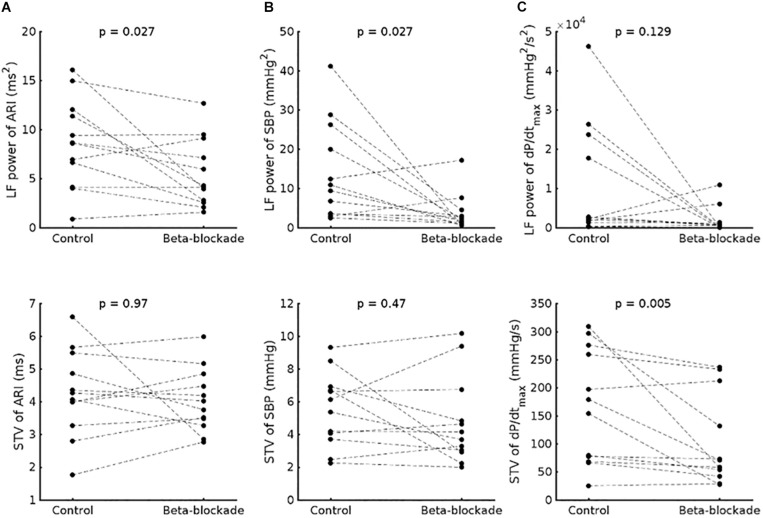
Effect of beta-adrenergic blockade on the low frequency (LF) power (top) and short-term variability (STV) (bottom) of **(A)** activation-recovery intervals (ARIs), **(B)** systolic blood pressure (SBP) and **(C)** the maximum rate of systolic pressure increase (dP/dt_max_).

In the group data, beta-adrenergic blockade resulted in a significant reduction of LF power of ARI (8.6 ± 4.5 ms^2^ vs. 5.5 ± 3.5 ms^2^, *p* = 0.027) (Figure 3A) and the LF power of SBP (1.4 × 10^–3^ ± 1.2 × 10^–3^ mmHg^2^ vs. 0.4 × 10^–3^ ± 0.5 × 10^–3^ mmHg^2^, *p* = 0.027) (Figure 3B). A trend to reduction was observed for the LF power of dP/dt_max_ (0.7 × 10^–6^ ± 1 × 10^–6^ vs. 0.1 × 10^–6^ ± 0.2 × 10^–6^ mmHg^2^/s^2^, *p* = 0.129) (Figure 3C).

No effect of beta-adrenergic blockade was seen on the HF power of ARI (6.5 × 10^–3^ ± 3 × 10^–3^ ms^2^ vs. 6.1 × 10^–3^ ± 3.4 × 10^–3^ ms^2^, *p* = 0.91), SBP (1.9 × 10^–3^ ± 1 × 10^–3^ mmHg^2^ vs. 1.7 × 10^–3^ ± 1 × 10^–3^ mmHg^2^, *p* = 0.424), nor dP/dt_max_ (7.6 × 10^–7^ ± 7.4 × 10^–7^ mmHg^2^/s^2^ vs. 3.2 ± 3.9 mmHg^2^/s^2^, *p* = 0.052).

No immediate effect of beta-adrenergic blockade was seen on mean ARI for group data (186.9 ± 22.8 vs. 186.5 ± 20.5 ms, *p* = 0.4) or the beat-to-beat variability (STV-ARI: 4.26 ± 1.3 vs. 4.03 ± 0.96 ms, *p* = 0.97). We also did not observe an effect on the beat-to-beat variability of SBP (STV-SBP: 5.52 ± 2.25 vs. 4.75 ± 2.68 mmHg, *p* = 0.380), but the STV dP/dt_max_ was significantly reduced (STV-dP/dt_max_ 166 ± 102 vs. 102 ± 80 mmHg/s, *p* = 0.005).

**FIGURE 4:**
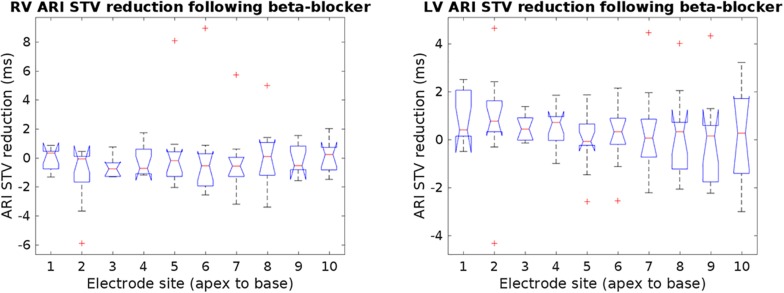
Reduction of ARI short-time variability (STV) following beta-blocker from individual electrodes in the right and left ventricle (RV and LV). No significant changes in STV reduction were found across electrode sites. Outliers are marked by crosses.

The ARI STV response to beta-adrenergic blockade was not different across breathing frequencies: mean ARI STV reduction −0.1 (±0.7) for 15 breaths/min versus 0.2 (±1.4) ms for 30 breaths/min, *p* = 0.8. Furthermore, as shown in Figure 4, there were no significant differences in ARI STV reduction between electrode sites in the RV and LV (*p* = 0.87 and *p* = 0.56 for RV and LV, respectively). We also tested the differences in STV baseline and reduction between RV and LV. Mean values of STV baseline and reduction were slightly higher in the LV, but the differences were not statistically significant: mean ARI STV baseline: 8.7 ± 8.1 vs. 9.3 ± 5.0 ms, *p* = 0.4; mean ARI STV reduction: −0.1 ± 1.8 vs. 0.3 ± 1.0, *p* = 0.1, for RV and LV, respectively. Finally, we also investigated whether the LF power and STV response to beta-adrenergic blockade was different in patients who had previously been treated with beta-blocker (*n* = 7, Table 1) compared to those who had not (*n* = 5). Although medication was discontinued for 5 days before the study in all patients, we found that the average response in both ARI LF power and ARI STV was slightly higher in patients treated with beta-blockers, but the numbers were too small to allow robust statistical analysis (mean reduction in LF power: 4.3 ± 5.3 vs. 1.5 ± 1.3 ms^2^; mean reduction in ARI STV reduction: 0.7 ± 1.5 vs. −0.4 ± 0.5 ms).

### Influence of Initial Values on the Response to Beta-Adrenergic Blockade

**FIGURE 5:**
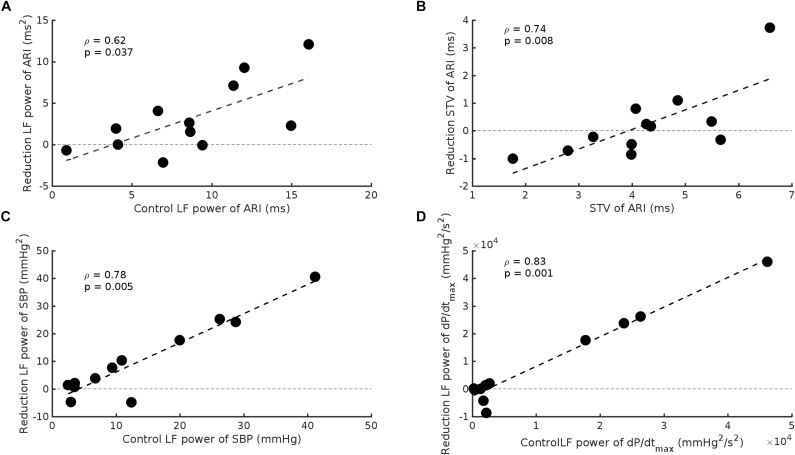
Scatterplots demonstrating the significant relationship between baseline values and the reduction seen following beta-adrenergic blockade in: **(A)** LF power of ARI, **(B)** beat-to-beat variability of ARI (STV ARI), **(C)** LF power of systolic blood pressure (SBP), and **(D)** the LF power of the maximum rate of systolic pressure increase (dP/dt_max_).

The effect of beta-adrenergic blockade on the group data was small. However, a wide range of control values was evident and when our results were expressed in relation to control values a highly significant effect of beta-adrenergic blockade was apparent. Subjects in whom the initial control values of LF of ARI were large showed a greater change in the magnitude of the oscillations following beta-adrenergic blockade compared to subjects in whom the initial values were low. When control oscillations of ARI were large beta-adrenergic blockade reduced their magnitude. When control oscillations were small the response to beta-adrenergic blockade was minimal or variable (*r*_s_ = 0.62, *p* = 0.037) (Figure 5A). A similar relationship was observed for ARI-STV (rs = 0.74, *p* = 0.008) (Figure 5B), and the LF power of SBP and dP/dt_max_ (*r*_s_ = 0.78, *p* = 0.004 and *r*_s_ = 0.84, *p* = 0.001, respectively) (Figures 5C,D).

### Relationship Between Low Frequency Power and Beat-to-Beat Variability of ARI

**FIGURE 6:**
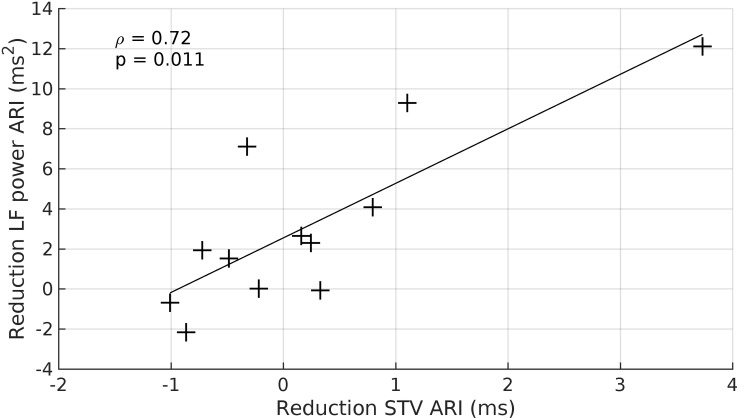
Scatterplot demonstrating the significant relationship between the beta-adrenergic blockade induced reduction in the LF power ARI and the witnessed reduction in beat-to-beat variability of ARI (STV ARI). +, data point.

There was a strong relationship between the reduction in LF power of ARI and the reduction of STV-ARI in response to beta-adrenergic blockade (rs = 0.72, *p* = 0.01) (Figure 6). No significant relationships were found between the reduction of LF power and SBP-STV (rs = 0.42, *p* = 0.2) or dP/dt_max_-STV (rs = 0.36, *p* = 0.3). There was also no significant relationships between the reduction in HF power and STV for ARI, SBP, and dP/dt_max_: (ARI-STV: rs = 0.48, *p* = 0.1. SBP-STV: rs = 0.38, *p* = 0.3; dP/dt_max_ –STV: rs = 0.56, *p* = 0.06).

## Discussion

We studied the effect of acute beta-adrenergic blockade on LF oscillations of ventricular APD (approximated by ARI) and on beat-to-beat APD variability. LF power and STV ARI measurements were made from 10 RV and 10 LV sites and then averaged in patients with normal ventricles. Cycle length was maintained constant with right ventricular pacing to eliminate confounding effects of cycle length dependency and breathing was controlled throughout the protocol at 0.25 and 0.5 Hz. Our main findings were: (1) we observed a wide variation of control values of LF power and beat-to-beat variability of ARI, SBP and dP/dt_max_; (2) beta-adrenergic blockade was associated with a significant reduction of LF power of ARI and SBP, (3) individually no clear impact of beta-adrenergic blockade on the beat-to-beat variability of ARI, SBP and dP/dt_max_ was demonstrated, however, (4) there was a strong correlation between the reduction seen in the LF power of ARI, SBP, and dP/dt_max_ following beta-adrenergic blockade, and the reduction in beat-to-beat variability.

Whereas oscillations in heart rate variability have long been recognized and the underlying mechanisms the subject of much debate (Parati et al., 2006), oscillations of ventricular APD at the low frequency have only relatively recently been identified (Hanson et al., 2014). These LF APD oscillations identified in humans using ARI recordings from the ventricular myocardium, are independent of variation in R-R interval and independent of respiration (Hanson et al., 2014). They frequently occur in association with LF oscillations in blood pressure (Mayer waves) (Julien, 2006). Oscillation of ventricular repolarization at the low frequency has recently been identified from the body surface ECG T-wave vector and these are also independent of R-R interval variability and respiration and are attributed to LF oscillation of ventricular APD (Rizas et al., 2014). When enhanced these oscillations are strongly predictive of arrhythmia and sudden cardiac death (Rizas et al., 2014, 2016, 2017, 2019; Hamm et al., 2017). The magnitude of both ARI and T-wave vector oscillations is increased during sympathetic stimulation (Rizas et al., 2014; Porter et al., 2017, 2018) and it has been suggested they may be related to the intrinsic low frequency oscillation of sympathetic nerve activity.

Enhanced beat-to-beat variability of repolarization, measured clinically as QT variability or experimentally as APD variability, is well known to predispose to malignant ventricular arrhythmias (Atiga et al., 1998; Haigney et al., 2004; Thomsen et al., 2004; Gallacher et al., 2007; Tereshchenko et al., 2009; Abi-Gerges et al., 2010; Hinterseer et al., 2010; Jacobson et al., 2011; Średniawa et al., 2012; Baumert et al., 2016). BVR has been shown to be enhanced by beta-adrenergic stimulation (Johnson et al., 2010, 2013; Porter et al., 2017). Paradoxically studies using beta-adrenergic blockade have shown a mixed response of BVR in QT interval measurements with either no change or an increase or decrease (Baumert et al., 2016). In this work we demonstrate firstly a strong dependence of the effect of beta blockade on initial conditions, and secondly a possible interaction between LF power and beat-to-beat variability of ARI. Importantly, we observed that changes in beat-to-beat variability were more enhanced following beta-adrenergic blockade when LF power was reduced. In contrary, small or no changes were observed in beat-to-beat variability in individuals for which the LF power was not modulated. Although these findings may have important mechanistic implications in this context, it should be noted that beat-to-beat variability and low frequency power are both measures of variability, hence the observed relationship between LF power and beat-to-beat variability could also be a mathematical consequence. Nevertheless, the results could provide an explanation on the conflicting results reported on the effect of beta-adrenergic blockade on beat-to-beat variability. Recently it has also been observed that the intrinsic beat-to-beat variation in APD also exhibits phasic variation at the low frequency, which is enhanced during increased sympathetic stimulation (Porter et al., 2018), which may further support a possible interaction between LF ARI and intrinsic beat-to-beat variation in ARI. Interestingly, preliminary data from this study shows that the response of LF ARI power and beat-to-beat variability following beta-adrenergic blockade were more pronounced in individuals that had previously been treated with beta-blockers. While the number were too small to draw any final conclusions, it might highlight a role of the dynamic nature of beta-adrenergic receptors, but it is also possible that these patients had a higher sympathetic tone during control. Future work will further investigate this finding.

The present work was conducted in patients with normal hearts and we cannot exclude the possibility that the relationships we observed may have been different in patients with arrhythmias. However, in this context the following observations in patients with arrhythmias are worth mention. In a recent study in heart failure patients ARI recordings as a measure of local action potential duration were obtained from the left ventricular epicardial lead of an implanted cardiac defibrillator device. 11 of 43 patients received appropriate shock treatment for sustained ventricular tachycardia or fibrillation, and ARI variability was significantly higher in these patients compared to patients who did not develop arrhythmia (Porter et al., 2019). In the present paper we observed a relationship between the initial BVR and the corresponding reduction following beta blockade. Consequently beta-adrenergic blockade may have a greater effect on BVR in individuals at increased arrhythmic risk compared to those at low risk.

Clinical conditions associated with high arrhythmia risk are commonly accompanied by adverse ventricular remodeling with downregulation of ionic currents and dysregulation of Ca2+handling and reduced repolarization reserve (Armoundas et al., 2001). Beta-adrenergic stimulation in the presence of reduced repolarization reserve (Iks block) has been shown to dramatically increase BVR and be proarrhythmic (Johnson et al., 2010). The importance of downregulation of IKs in promoting excess BVR during beta-adrenergic stimulation was further demonstrated in a study identifying a role of calcium mediated mechanisms in the generation of arrhythmias (Johnson et al., 2013). In a recent study in the chronic AV block dog model using monophasic action potential recordings, remodeling resulted in an increase of low frequency oscillations of ventricular MAP duration. Furthermore, low frequency BVR measured as beat to beat differences of MAP duration, also increased. Increased low frequency power was positively related to Torsades de Pointes inducibility (Sprenkeler et al., 2019). These results suggest an interaction between the remodeling process, low frequency oscillation and beat to beat variability of ventricular repolarization as playing an important role in arrhythmogenesis. The findings are in keeping with recent computational modeling studies involving phasic low frequency beta-adrenergic and mechanical stimulation. When remodeling was simulated by reducing repolarization reserve (reduced Ikr) and incorporating calcium overload early after depolarizations and triggered activity were readily induced (Pueyo et al., 2016b; Sampedro-Puente et al., 2019).

While a number of studies have investigated the cellular mechanisms underlying modulation of BVR by beta-adrenergic stimulation and the consequent effect on arrhythmia initiation (Johnson et al., 2010, 2013; Szentandrássy et al., 2015), only a few have so far examined mechanisms underlying low frequency oscillation of ventricular APD (Pueyo et al., 2016b; Sampedro-Puente et al., 2019). Regarding BVR ion channel stochasticity and calcium cyclical variation have both been identified as major contributors. Regarding low frequency oscillations of ventricular APD a direct action of beta-adrenergic stimulation and mechano-electric feedback has been suggested.

Recent computational research has shown that the major ionic contributors to inter-individual differences in LF oscillations of APD and beat-to-beat APD variability are I_Kr_, I_CaL_, and I_K__1_ (Sampedro-Puente et al., 2019). In this study, a set of stochastic human ventricular action potential models was developed by individually varying the ionic conductances of I_Kr_, I_CaL_, and I_K__1_ from their nominal values in the O’Hara-Virág-Varró-Rudy (ORd) action potential model (O’Hara et al., 2011). Beta-adrenergic and mechanical stretch effects were included in the models to simulate sympathetic modulation of ventricular electrophysiology at the cell level (Pueyo et al., 2016b; Sampedro-Puente et al., 2019). For each of the simulated models, normalized measures of LF oscillation magnitude of APD (nmLF) and beat-to-beat APD variability (STV-APD) were computed before and after beta-adrenergic blockade. In accordance with the clinical observations of this study, beta-adrenergic blockade in these simulated cells led to a remarkable reduction in nmLF and also in STV-APD. Importantly, these simulations showed a wide range of nmLF and STV-APD initial values as well as of their changes in response to beta-adrenergic blockade. In line with the presented clinical data, higher nmLF and STV-APD initial values were associated with larger beta-adrenergic blockade-induced decreases in the magnitudes of both markers. A strong correlation was observed between the effects of beta-adrenergic blockade on nmLF and STV-APD.

The reduction in nmLF in response to beta-adrenergic blockade, which could be observed to a greater or lesser extent in all the virtual cells, can be explained on the basis of beta-adrenergic stimulation enhancing LF oscillations of APD via differential phosphorylation and dephosphorylation kinetics of cellular PKA targets (mainly I_CaL_ and I_Ks_) (Pueyo et al., 2016b; Sampedro-Puente et al., 2019). For STV-APD, the reduction induced by beta-adrenergic blockade is justified by the fact that beta-adrenergic stimulation modulates, on the one hand, the LF oscillations of APD and, on the other hand, the stochastic gating of ionic currents active during the repolarization phase (Sampedro-Puente et al., 2019).

Mechanoelectric feedback (MEF) has been suggested to contribute to the development of LF oscillations and BVR of APD in humans *in vivo* (Hanson et al., 2014) and by computational simulation, these adrenergic and mechanical actions have been shown to synergistically potentiate the oscillatory behavior and temporal variability of cellular ventricular repolarization (Pueyo et al., 2016b; Sampedro-Puente et al., 2019), in accord with the well-known potentiation of MEF effects by beta-adrenergic stimulation (Horner et al., 1996; Puglisi et al., 2013). The role of MEF, possibly through stretch-activated channels, in contributing to BVR is supported by experimental evidence in the chronic atrioventricular-block dog model, where beat-to-beat preload changes have been shown to increase short-term variability of monophasic APD (Stams et al., 2016). The timing of electro-mechanical coupling may also be important. In a canine model IKs block prolonged APD altering the timing of ventricular repolarization in relation to the ventricular pressure curve. Under these conditions the addition of left stellate stimulation induced Torsades de Points (ter Bekke et al., 2019).

The importance of risk stratification for arrhythmia and sudden death to guide patient selection for ICD implantation has already been stressed. A number of non-invasive markers of risk have been proposed including amongst others heart rate variability, baroreflex sensitivity, microvolt T-wave alternans, heart rate turbulence, Tpeak-Tend as an index of dispersion of repolarization and QT interval variability, all of which are modulated by autonomic activity (Baumert et al., 2016; Priori et al., 2016; Tse et al., 2017). However, despite showing promise none of these has so far influenced clinical practice. Numerous studies have examined the predictive power of BVR estimated in humans as QT variability or intracardiac QT interval as have been comprehensively summarized by Baumert et al. (2016). While many studies showed encouraging results a significant number were less so. It was concluded by these authors that analysis of joint RR and QT dynamics seems to allow detecting repolarization stability preceding malignant ventricular arrhythmias in patients post MI, and prospective studies are needed on the predictive value of QTV as part of a multivariate risk stratification procedure in different well-defined populations. The variable that has shown the most consistent association with sudden cardiac death is reduced left ventricular ejection fraction and remains the gold standard for risk stratification of patients with ischemic heart disease and primary prevention (Priori et al., 2016). A conceptually attractive aspect of the application of BVR is that experimental work in a canine complete AV block model indicates that the strong association with inducible TdP/VF reflects ventricular remodeling which is a characteristic feature of at-risk patients (Smoczynska et al., 2019). A recent multicenter prospective clinical trial involving 44 centers in 15 EU countries now provides convincing evidence for enhanced low frequency oscillations of ventricular repolarization, measured from the ECG T-wave vector referred to as Periodic Repolarization Dynamics (PRD), to be one of the strongest predictors of ventricular arrhythmia and sudden death in post MI patients (Bauer et al., 2019). Comparison of the potential clinical value of each of these various biomarkers is hindered by the fact that most studies have focused on just one or a small number of these parameters and the lack of any standardization of methodology and study population. Future research should focus on evaluating the prognostic value of possible combinations of these biomarkers in prospective multivariate analysis in specific patient populations.

### Limitations

The study population were patients with ostensibly normal ventricles undergoing routine ablation procedures for supraventricular arrhythmias. Eight of the 12 patients had persistent atrial fibrillation and therefore the possibility of some ventricular remodeling cannot be excluded. However, the routine procedure for atrial fibrillation ablation involves transseptal puncture to allow access to the left atrium from the right atrium. This enables placement of an LV decapolar catheter for the research procedure (right atrium to left atrium to left ventricle via the mitral valve) without the need for arterial puncture for retrograde access to the left ventricle. In many years experience of acquiring basic electrophysiological data from the *in vivo* human heart in order to complement laboratory studies, we have always considered it a priority to integrate the research protocol with the clinical protocol avoiding additional invasive procedures. We recognize that it would be ideal to have longer recordings when studying LF related parameters, but to comply with clinical studies we designed the study with view to limiting the duration of the study as much as possible.

Recordings were made from 20 localized right and left ventricular endocardial sites and then averaged. It is possible that other regions may have yielded different results. Furthermore, averaging may confound local beat-to-beat variabilities, although we did not find evidence for this when comparing the STV reduction across electrode sites and between left and right ventricle. In addition, breathing frequency may also play a role. In this work we report averaged data from two different breathing frequencies (15 and 30 breaths/min), but the reduction in ARI STV between the two breathing frequencies was not found to be significantly different.

### Clinical Implications

Understanding the mechanisms underlying the interaction between beta-adrenergic stimulation, the LF oscillatory behavior of APD and beat-to-beat APD variability is important for the development of therapeutic strategies for the prevention of arrhythmia and sudden cardiac death. Enhanced oscillations of ventricular repolarization in the LF range measured from the ECG T-wave vector and referred to as periodic repolarization dynamics (PRD) have emerged as one of the strongest predictors of arrhythmia and sudden cardiac death in cardiac patients and are the subject of ongoing clinical trials (Rizas et al., 2014, 2016, 2017, 2019; Hamm et al., 2017; Bauer et al., 2019). The present work identifies several specific features of the interaction between beta-adrenergic stimulation, the LF oscillatory behavior of APD and beat-to-beat APD variability that are reproducible by computational modeling which enables mechanistic insight to be gained at the cellular level.

## Conclusion

In patients with normal ventricles acute beta-adrenergic blockade modulated LF oscillatory behavior of ventricular APD (measured as ARIs) and beat-to-beat variability of APD in a manner that was dependent on baseline APD variability. A strong correlation was present between the effect of beta-adrenergic blockade on LF oscillation of APD and beat-to-beat variability of APD. These findings are discussed in relation to computational modeling which reproduced the clinical findings and investigated cellular mechanisms. These observations provide valuable insight into the strong association of LF oscillations of ventricular repolarization and arrhythmic and sudden cardiac death. Further work is warranted to improve our understanding in order to develop therapeutic strategies.

## Data Availability Statement

The datasets generated for this study are available on request to the corresponding author.

## Ethics Statement

The studies involving human participants were reviewed and approved by the Ethics Committee of Guy’s and Thomas’ Hospitals. The patients/participants provided their written informed consent to participate in this study.

## Author Contributions

BP, SV, JG, and PT conceived and designed the experiments. All authors took responsibility in analyzing and interpreting the data, contributed to drafting or revising the manuscript, and approved the final version of the manuscript.

## Conflict of Interest

The authors declare that the research was conducted in the absence of any commercial or financial relationships that could be construed as a potential conflict of interest.

## References

[B1] Abi-Gerges N. Valentin J. P. Pollard C. E. (2010). Dog left ventricular midmyocardial myocytes for assessment of drug-induced delayed repolarization: short-term variability and proarrhythmic potential. *Br. J. Pharmacol.* 159 77–92. 10.1111/j.1476-5381.2009.00338.x 19663882 PMC2823354

[B2] Armoundas A. A. Wu R. Juang G. Marbán E. Tomaselli G. F. (2001). Electrical and structural remodeling of the failing ventricle. *Pharmacol. Ther.* 92 213–230. 10.1016/S0163-7258(01)00171-171 11916538

[B3] Atiga W. L. Calkins H. Lawrence J. H. Tomaselli G. F. Smith J. M. Berger R. D. (1998). Beat-to-beat repolarization lability identifies patients at risk for sudden cardiac death. *J. Cardiovasc. Electrophysiol.* 9 899–908. 10.1111/j.1540-8167.1998.tb00130.x 9786070

[B4] Bauer A. Klemm M. Rizas K. D. Hamm W. von Stülpnagel L. Dommasch M. (2019). Prediction of mortality benefit based on periodic repolarisation dynamics in patients undergoing prophylactic implantation of a defibrillator: a prospective, controlled, multicentre cohort study. *Lancet* 394 1344–1351. 10.1016/S0140-6736(19)31996-8 31488371

[B5] Baumert M. Porta A. Vos M. A. Malik M. Couderc J. P. Laguna P. (2016). QT interval variability in body surface ECG: measurement, physiological basis, and clinical value: position statement and consensus guidance endorsed by the European heart rhythm association jointly with the ESC working group on cardiac cellular electroph. *Europace* 18 925–944. 10.1093/europace/euv405 26823389 PMC4905605

[B6] Coronel R. de Bakker J. M. T. Wilms-Schopman F. J. G. Opthof T. Linnenbank A. C. Belterman C. N. (2006). Monophasic action potentials and activation recovery intervals as measures of ventricular action potential duration: experimental evidence to resolve some controversies. *Hear. Rhythm* 3 1043–1050. 10.1016/j.hrthm.2006.05.027 16945799

[B7] Gallacher D. J. Van de Water A. van der Linde H. Hermans A. N. Lu H. R. Towart R. (2007). In vivo mechanisms precipitating torsades de pointes in a canine model of drug-induced long-QT1 syndrome. *Cardiovasc. Res.* 76 247–256. 10.1016/j.cardiores.2007.06.019 17669388

[B8] Haigney M. C. Zareba W. Gentlesk P. J. Goldstein R. E. Illovsky M. McNitt S. (2004). QT interval variability and spontaneous ventricular tachycardia or fibrillation in the Multicenter Automatic Defibrillator Implantation Trial (MADUT) II patients. *J. Am. Coll. Cardiol.* 44 1481–1487. 10.1016/j.jacc.2004.06.063 15464332

[B9] Hamm W. Rizas K. D. Stülpnagel L. V. Vdovin N. Massberg S. Kääb S. (2017). Implantable cardiac monitors in high-risk post-infarction patients with cardiac autonomic dysfunction and moderately reduced left ventricular ejection fraction: design and rationale of the SMART-MI trial. *Am. Heart J.* 190 34–39. 10.1016/j.ahj.2017.05.006 28760211

[B10] Hanson B. Child N. Van Duijvenboden S. Orini M. Chen Z. Coronel R. (2014). Oscillatory behavior of ventricular action potential duration in heart failure patients at respiratory rate and low frequency. *Front. Physiol.* 5:414 10.3389/fphys.2014.00414 PMC4211392 25389408

[B11] Hanson B. Gill J. Western D. Gilbey M. P. Bostock J. Boyett M. R. (2012). Cyclical modulation of human ventricular repolarization by respiration. *Front. Physiol.* 3:379. 10.3389/fphys.2012.00379 23055983 PMC3457072

[B12] Haws C. W. Lux R. L. (1990). Correlation between in vivo transmembrane action potential durations and activation-recovery intervals from electrograms. Effects of interventions that alter repolarization time. *Circulation* 81 281–288. 10.1161/01.CIR.81.1.281 2297832

[B13] Hinterseer M. Beckmann B. M. Thomsen M. B. Pfeufer A. Ulbrich M. Sinner M. F. (2010). Usefulness of short-term variability of QT intervals as a predictor for electrical remodeling and proarrhythmia in patients with nonischemic heart failure. *Am. J. Cardiol.* 106 216–220. 10.1016/j.amjcard.2010.02.033 20599006

[B14] Horner S. M. Murphy C. F. Coen B. Dick D. J. Lab M. J. (1996). Sympathomimetic modulation of load-dependent changes in the action potential duration in the in situ porcine heart. *Cardiovasc. Res.* 32 148–157. 10.1016/0008-6363(96)00087-9 8776412

[B15] Jacobson I. Carlsson L. Duker G. (2011). Beat-by-beat QT interval variability, but not QT prolongation per se, predicts drug-induced torsades de pointes in the anaesthetised methoxamine-sensitized rabbit. *J. Pharmacol. Toxicol. Methods* 63 40–46. 10.1016/j.vascn.2010.04.010 20451633

[B16] Johnson D. M. Heijman J. Bode E. F. Greensmith D. J. Van Der Linde H. Abi-Gerges N. (2013). Diastolic spontaneous calcium release from the sarcoplasmic reticulum increases beat-to-beat variability of repolarization in canine ventricular myocytes after β-adrenergic stimulation. *Circ. Res.* 112 246–256. 10.1161/CIRCRESAHA.112.275735 23149594

[B17] Johnson D. M. Heijman J. Pollard C. E. Valentin J. P. Crijns H. J. G. M. Abi-Gerges N. (2010). IKs restricts excessive beat-to-beat variability of repolarization during beta-adrenergic receptor stimulation. *J. Mol. Cell. Cardiol.* 48 122–130. 10.1016/j.yjmcc.2009.08.033 19744496

[B18] Julien C. (2006). The enigma of mayer waves: facts and models. *Cardiovasc. Res.* 70 12–21. 10.1016/j.cardiores.2005.11.008 16360130

[B19] Monge Garcia M. I. Jian Z. Settels J. J. Hunley C. Cecconi M. Hatib F. (2018). Performance comparison of ventricular and arterial dP/dtmax for assessing left ventricular systolic function during different experimental loading and contractile conditions. *Crit. Care* 22:325. 10.1186/s13054-018-2260-1 30486866 PMC6262953

[B20] O’Hara T. Virág L. Varró A. Rudy Y. (2011). Simulation of the undiseased human cardiac ventricular action potential: model formulation and experimental validation. *PLoS Comput. Biol.* 7:e1002061. 10.1371/journal.pcbi.1002061 21637795 PMC3102752

[B21] Parati G. Mancia G. Di Rienzo M. Castiglioni P. Taylor J. Studinger P. (2006). Point: counterpoint point: counterpoint: cardiovascular variability is / is not an index of autonomic control of circulation. *J. Appl. Physiol.* 101 676–688. 10.1152/japplphysiol.00446.2006.Point 16645191

[B22] Porter B. Bishop M. J. Claridge S. Behar J. Sieniewicz B. J. Webb J. (2017). Autonomic modulation in patients with heart failure increases beat-to-beat variability of ventricular action potential duration. *Front. Physiol.* 8:328. 10.3389/fphys.2017.00328 28611676 PMC5447044

[B23] Porter B. Bishop M. J. Claridge S. Child N. Van Duijvenboden S. Bostock J. (2019). Left ventricular activation-recovery interval variability predicts spontaneous ventricular tachyarrhythmia in patients with heart failure. *Hear. Rhythm* 16 702–709. 10.1016/j.hrthm.2018.11.013 30528448

[B24] Porter B. Van Duijvenboden S. Bishop M. J. Orini M. Claridge S. Gould J. (2018). Beat-to-Beat variability of ventricular action potential duration oscillates at low frequency during sympathetic provocation in humans. *Front. Physiol.* 9:147. 10.3389/fphys.2018.00147 29670531 PMC5893843

[B25] Potse M. Vinet A. Opthof T. Coronel R. (2009). Validation of a simple model for the morphology of the T wave in unipolar electrograms. *Am. J. Physiol. Heart Circ. Physiol.* 297 H792–H801. 10.1152/ajpheart.00064.2009 19465555

[B26] Priori S. G. Blomström-Lundqvist C. Mazzanti A. Blom N. Borggrefe M. Camm J. (2016). 2015 ESC guidelines for the management of patients with ventricular arrhythmias and the prevention of sudden cardiac death. *Russ. J. Cardiol.* 7 5–86. 10.1016/j.rec.2016.01.001 26837728

[B27] Pueyo E. Dangerfield C. E. Britton O. J. Virág L. Kistamás K. Szentandrássy N. (2016a). Experimentally-based computational investigation into beat-to-beat variability in ventricular repolarization and its response to ionic current inhibition. *PLoS One* 11:e0151461. 10.1371/journal.pone.0151461 27019293 PMC4809506

[B28] Pueyo E. Orini M. Rodríguez J. F. Taggart P. (2016b). Interactive effect of beta-adrenergic stimulation and mechanical stretch on low-frequency oscillations of ventricular action potential duration in humans. *J. Mol. Cell. Cardiol.* 97 93–105. 10.1016/j.yjmcc.2016.05.003 27178727

[B29] Puglisi J. L. Negroni J. A. Chen-Izu Y. Bers D. M. (2013). The force-frequency relationship: insights from mathematical modeling. *Adv. Physiol. Educ.* 37 28–34. 10.1152/advan.00072.2011 23471245 PMC3776472

[B30] Rizas K. D. Doller A. J. Hamm W. Vdovin N. von Stuelpnagel L. Zuern C. S. (2019). Periodic repolarization dynamics as risk predictor after myocardial infarction: prospective validation study. *Hear. Rhythm* 16 1223–1231. 10.1016/j.hrthm.2019.02.024 30818092

[B31] Rizas K. D. Hamm W. Kääb S. Schmidt G. Bauer A. (2016). Periodic repolarisation dynamics: a natural probe of the ventricular response to sympathetic activation. *Arrhythmia Electrophysiol. Rev.* 5 31–36. 10.15420/aer.2015 27403291 PMC4939306

[B32] Rizas K. D. McNitt S. Hamm W. Massberg S. Kääb S. Zareba W. (2017). Prediction of sudden and non-sudden cardiac death in post-infarction patients with reduced left ventricular ejection fraction by periodic repolarization dynamics: MADIT-II substudy. *Eur. Heart J.* 38 2110–2118. 10.1093/eurheartj/ehx161 28431133 PMC5837472

[B33] Rizas K. D. Nieminen T. Barthel P. Zürn C. S. Kähönen M. Viik J. (2014). Clinical medicine Sympathetic activity – associated periodic repolarization dynamics predict mortality following myocardial infarction. *J. Clin. Invest.* 124 1770–1780. 10.1172/JCI70085DS1 24642467 PMC3973112

[B34] Sampedro-Puente D. A. Fernandez-Bes J. Porter B. van Duijvenboden S. Taggart P. Pueyo E. (2019). Mechanisms underlying interactions between low-frequency oscillations and beat-to-beat variability of celullar ventricular repolarization in response to sympathetic stimulation: implications for arrhythmogenesis. *Front. Physiol.* 10:916. 10.3389/fphys.2019.00916 31427979 PMC6687852

[B35] Smoczynska A. Beekman H. D. Vos M. A. (2019). The increment of short-term variability of repolarisation determines the severity of the imminent arrhythmic outcome. *Arrhythmia Electrophysiol. Rev.* 8 166–172. 10.15420/aer.2019.16.2 31576205 PMC6766692

[B36] Sprenkeler D. J. Beekman J. D. M. Bossu A. Dunnink A. Vos M. A. (2019). Pro-Arrhythmic ventricular remodeling is associated with increased respiratory and low-frequency oscillations of monophasic action potential duration in the chronic atrioventricular block dog model. *Front. Physiol.* 10:1095. 10.3389/fphys.2019.01095 31507455 PMC6716537

[B37] Średniawa B. Kowalczyk J. Lenarczyk R. Kowalski O. Sȩdkowska A. Cebula S. (2012). Microvolt T-wave alternans and other noninvasive predictors of serious arrhythmic events in patients with an implanted cardioverter-defibrillator. *Kardiol. Pol.* 70 447–455. 22623232

[B38] Stams T. R. G. Oosterhoff P. Heijdel A. Dunnink A. Beekman J. D. M. van der Nagel R. (2016). Beat-to-beat variability in preload unmasks latent risk of Torsade de Pointes in anesthetized chronic atrioventricular block dogs. *Circ. J.* 80 1336–1345. 10.1253/circj.CJ-15-1335 27151565

[B39] Szentandrássy N. Kistamás K. Hegyi B. Horváth B. Ruzsnavszky F. Váczi K. (2015). Contribution of ion currents to beat-to-beat variability of action potential duration in canine ventricular myocytes. *Pflugers Arch. Eur. J. Physiol.* 467 1431–1443. 10.1007/s00424-014-1581-4 25081243

[B40] ter Bekke R. M. A. Moers A. M. E. de Jong M. M. J. Johnson D. M. Schwartz P. J. Vanoli E. (2019). Proarrhythmic proclivity of left-stellate ganglion stimulation in a canine model of drug-induced long-QT syndrome type 1. *Int. J. Cardiol.* 286 66–72. 10.1016/j.ijcard.2019.01.098 30777408

[B41] Tereshchenko L. G. Fetics B. J. Berger R. D. (2009). Intracardiac QT variability in patients with structural heart disease on class III antiarrhythmic drugs. *J. Electrocardiol.* 42 505–510. 10.1016/j.jelectrocard.2009.07.011 19700170

[B42] Thomsen M. B. Verduyn S. C. Stengl M. Beekman J. D. M. De Pater G. Van Opstal J. (2004). Increased short-term variability of repolarization predicts d-sotalol-induced torsades de pointes in dogs. *Circulation* 110 2453–2459. 10.1161/01.CIR.0000145162.64183.C8 15477402

[B43] Thomson D. J. (1982). Spectrum estimation and harmonic analysis. *Proc. IEEE* 70 1055–1096. 10.1109/PROC.1982.12433

[B44] Tse G. Gong M. Wong W. T. Georgopoulos S. Letsas K. P. Vassiliou V. S. (2017). The tpeak - tend interval as an electrocardiographic risk marker of arrhythmic and mortality outcomes: a systematic review and meta-analysis. *Hear. Rhythm* 14 1131–1137. 10.1016/j.hrthm.2017.05.031 28552749

[B45] van Duijvenboden S. Hanson B. Child N. Orini M. Rinaldi C. A. Gill J. S. (2015). Effect of autonomic blocking agents on the respiratory-related oscillations of ventricular action potential duration in humans. *Am. J. Physiol. Heart Circ. Physiol.* 309 H2108–H2117. 10.1152/ajpheart.00560.2015 26475587 PMC4698427

[B46] Wyatt R. F. Burgess M. J. Evans A. K. Lux R. L. Abildskov J. A. Tsutsumi T. (1981). Estimation of ventricular transmembrane action potential durations and repolarization times from unipolar electrograms. *Am. J. Cardiol.* 47:488 10.1016/0002-9149(81)91028-6

